# Carboxymethyl-γ-cyclodextrin, a novel selective relaxant binding agent for the reversal of neuromuscular block induced by aminosteroid neuromuscular blockers: an ex vivo laboratory study

**DOI:** 10.1186/s12871-021-01424-4

**Published:** 2021-08-17

**Authors:** Ákos I. Fábián, Edömér Tassonyi, Vera Csernoch, Marianna Fedor, Tamás Sohajda, Lajos Szente, Béla Fülesdi

**Affiliations:** 1 Department of Anaesthesiology and Intensive Care, University of Debrecen Clinical Center, Nagyerdei krt. 98, 4012 Debrecen, Hungary; 2 Cyclolab Ltd, Budapest, Hungary

**Keywords:** Neuromuscular blocking agent, Selective relaxant binding agent, Cyclodextrin, Sugammadex, Rocuronium, Pipecuronium, Vecuronium, Phrenic nerve, Hemidiaphragm

## Abstract

**Background:**

Residual neuromuscular block at the end of surgery may compromise the patient’s safety. The risk of airway complications can be minimized through monitoring of neuromuscular function and reversal of neuromuscular block if needed. Effective reversal can be achieved with selective relaxant binding agents, however, sugammadex is the only clinically approved drug in this group.

We investigated the concentration–response properties of a novel selective relaxant binding agent, carboxymethyl-γ-cyclodextrin for the reversal of neuromuscular block. We evaluated the hypothesis that it is equally potent for reversing neuromuscular block as sugammadex.

**Methods:**

Phrenic nerve – hemidiaphragm tissue preparations were isolated from male Wistar rats and suspended in a tissue holder allowing electrical stimulation of the nerve and monitoring of muscle contraction force. Concentration–response relationships were constructed for the neuromuscular blocking agents rocuronium, pipecuronium, and vecuronium. The half-effective concentrations of sugammadex and carboxymethyl-γ-cyclodextrin for reversal of neuromuscular block were determined.

**Results:**

The half effective concentrations (95% confidence interval, CI) were 7.50 (6.93–8.12) μM for rocuronium, 1.38 (1.33–1.42) μM for pipecuronium, and 3.69 (3.59–3.80) μM for vecuronium. The half effective concentrations (95% CI) of carboxymethyl-γ-cyclodextrin and sugammadex were 35.89 (32.67–39.41) μM and 3.67 (3.43–3.92) μM, respectively, for the reversal of rocuronium-induced block; 10.14 (9.61–10.70) μM and 0.67 (0.62–0.74) μM, respectively, for the reversal of pipecuronium-induced block; and 376.1 (341.9–413.8) μM and 1.45 (1.35–1.56) μM, respectively, for the reversal of vecuronium-induced block.

**Conclusions:**

Carboxymethyl-γ-cyclodextrin is an effective, but less potent agent for reversal of neuromuscular block than sugammadex.

## Background

The safe use of non-depolarizing neuromuscular blocking agents (NMBA) requires their rapid and reliable reversal from any depth of block following their administration, without the risk of postoperative re-paralysis or other sequelae. Neostigmine has been used for 80 years as a reversal agent without fulfilling these requirements [1, 2]. A paradigm shift in clinical practice occurred when sugammadex, a modified γ-cyclodextrin derivative emerged in 2008 as a selective binding agent of free rocuronium molecules in the plasma [3–6]. Sugammadex was able to rapidly reverse any depth of rocuronium-induced neuromuscular block within 3 to 5 min, depending on its dose [7, 8]. Subsequent clinical trials showed reversal with sugammadex to be superior to neostigmine [9–11] and also effective in the reversal of pipecuronium-[12] and vecuronium-induced block [13, 14].

Sugammadex has generally been a success story, however, several limitations remain. Sugammadex is ineffective against benzylisoquinoline compounds [15]. Although generally safe, sugammadex can rarely cause clinically severe side effects [16]. Therefore research is ongoing to find alternatives to sugammadex. An effective alternative should ideally improve on the mentioned limitations while at the same time retaining similar in vivo efficacy for reversing neuromuscular block from aminosteroid NMBAs.

**Fig. 1:**
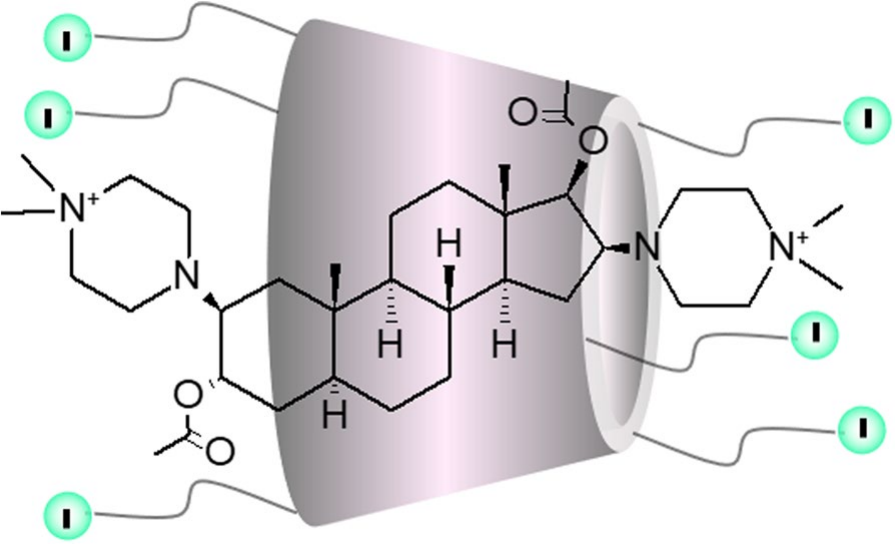
Schematic model of carboxymethyl-γ-cyclodextrin and the inclusion complex with neuromuscular blocking agents

To this end, in the present study, we evaluated the potency of a novel selective relaxant binding agent (SRBA), carboxymethyl-γ-cyclodextrin (CMGCD; Fig. 1). CMGCD is a γ-cyclodextrin derivative, which can have varying numbers of carboxymethyl sidechain substitutions (degree of substitution, DS) [17]. CMGCD was designed to form an inclusion complex with aminosteroid NMBAs. CMGCD’s potency for reversing rocuronium-, pipecuronium-, and vecuronium-induced block was measured in an ex vivo animal model and compared to sugammadex.

## Methods

### Animals

Ethics approval for this study (1/2013/DE MÁB) was provided by the University of Debrecen Committee of Animal Research, Debrecen, Hungary (Chairperson Prof I. Furka) on 15 April 2013. A total of 20 male Wistar rats ranging in weight from 250 to 563 g were used. Institutional guidelines for animal care and usage for research principles were strictly followed. Animals were chosen randomly on the morning of the experiment and killed before harvesting tissue specimens.

### Materials

Rocuronium (Esmeron; MSD Pharma Hungary, Budapest, Hungary), pipecuronium (Arduan; Richter Gedeon, Budapest, Hungary), vecuronium (Vecuronium Inresa; Inresa Arzneimittel Ltd, Freiburg, Germany), and sugammadex (Bridion; MSD Pharma Hungary, Budapest, Hungary) were purchased from commercial vendors and diluted in Krebs-buffer as needed to achieve a dosing volume of 10–100 μl.

CMGCD was developed and manufactured by Cyclolab Ltd, Budapest, Hungary. A detailed description of the synthesis process has been described [17]. CMGCD variants differ in their degree of substitution (DS). Based on preliminary ex vivo data, a CMGCD variant with DS = 4.1 was used for our experiments.

### Experimental procedures

The rat phrenic nerve-hemidiaphragm system was used for our experiments. Rats were killed with an intraperitoneal overdose of sodium thiopental (60 mg/kg) and exsanguinated through the incision of the dorsal vena cava. Hemidiaphragm preparation was performed by using a modified version of the technique originally described by Bülbring [18]. Briefly, bilateral thoracotomy and removal of the sternum were performed, after which both phrenic nerves were dissected from cranial to rostral direction to the diaphragm insertion. Then both hemidiaphragms were excised with the corresponding phrenic nerve intact. The hemidiaphragms were then secured in a phrenic nerve-diaphragm tissue holder (ISO-07-TSZ2D, Experimetria Ltd., Budapest, Hungary) in 75 mL of Krebs-puffer (110 mM NaCl, 5 mM KCl, 1.25 mM CaCl_2_, 1 mM MgSO_4_, 1 mM KH_2_PO_4_, 5 mM glucose, 20 mM NaHCO_3_) aerated by bubbling 95% O_2_ + 5% CO_2_ (Vol%) through the solution. The solution was maintained at a temperature of 37 °C (AMP-09 Temperature controller, Experimetria Ltd., Budapest, Hungary).

The hemidiaphragms were attached to an isometric force–displacement transducer (FSG-01/200 Force Transducer, Experimetria Ltd., Budapest, Hungary) at the diaphragmatic centrum tendineum using commercially available 5/0 diameter surgical suture. Measurements were amplified by an AMP-01-SG Classic bridge amplifier and recorded with a 16-channel professional software package (S.P.E.L. Advanced Isosys software, Experimetria Ltd., Budapest, Hungary). The phrenic nerve was stimulated either with a single twitch every 5 s (rectangular pulses of 0.3 ms pulse-width and supramaximal voltage) or a 2-Hz train-of-four (TOF) stimulus every 15 s (rectangular pulses of 0.2 ms duration with a supramaximal voltage) using a square wave stimulator (ST-03-O4, Experimetria Ltd., Budapest, Hungary).

**Fig. 2:**
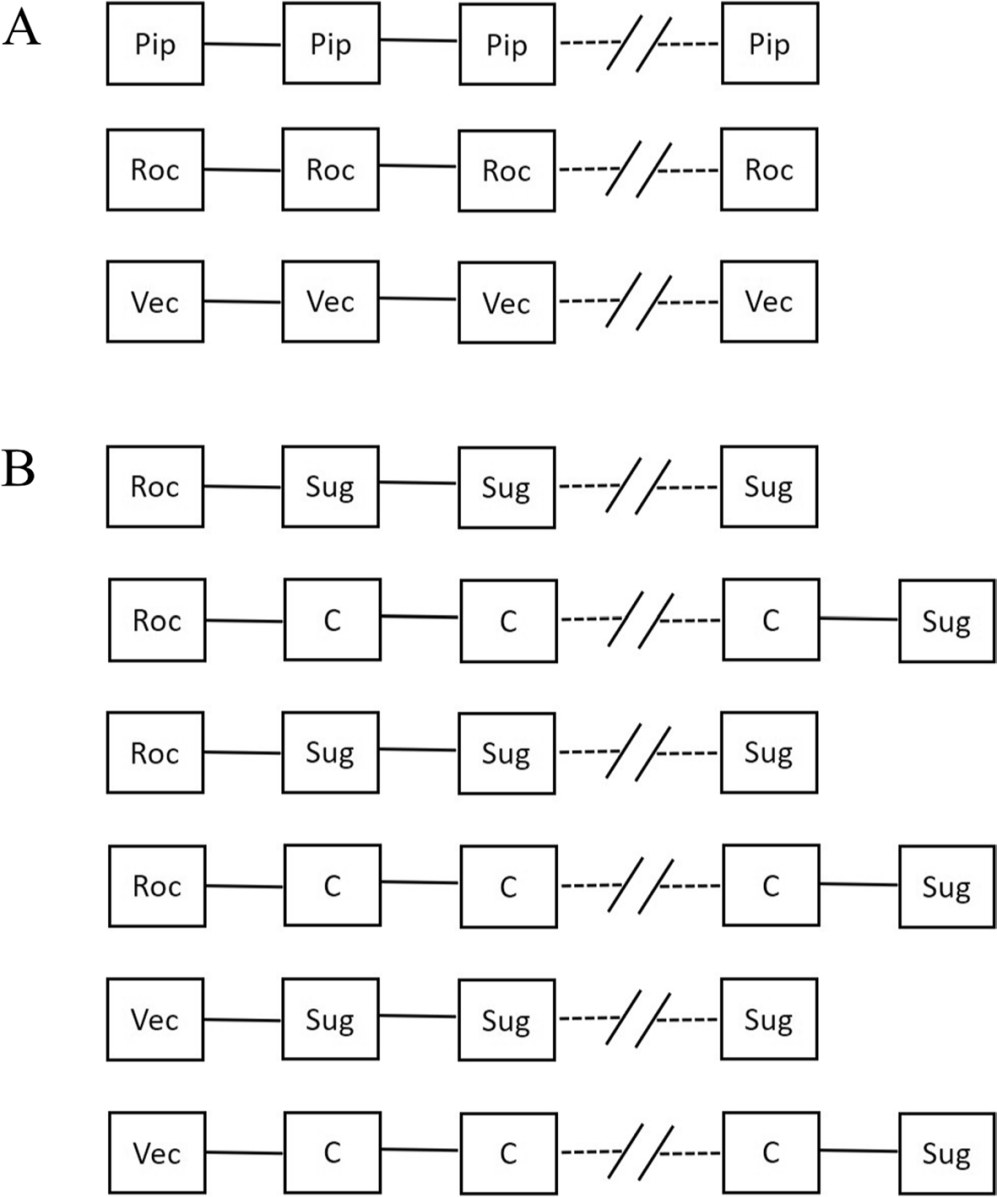
Study design. **A** Concentration–response curves for NMBAs. A bolus of NMBA was given every 15 min until the suppression of ST response was achieved. **B** Concentration–response curves for SRBAs. After an initial bolus of NMBA to achieve 90–95% depression of ST force amplitude, SRBA boluses were given every 15 min until full reversal of neuromuscular block was achieved. A reversal dose of sugammadex was given after reversal with C to verify full reversal. NMBA: neuromuscular blocking agent; ST: single twitch; SRBA: selective relaxant binding agent; Pip: pipecuronium; Roc: rocuronium; Vec: vecuronium; Sug: sugammadex; C: carboxymethyl-γ-cyclodextrin

After submersion in the buffer solution, the tissue preparations were allowed an acclimatization period of 10 min without stimulation at an applied resting tension of 20–30 mN. Then, stimulation was started followed by an additional 1–1.5 h without treatment (with buffer changes as needed) until a stable baseline tension was achieved. NMBA and SRBA dosing was only commenced after this stabilization period (for a scheme of drug dosing and study design, see Fig. 2). After the measurement of a given concentration–response curve, the buffer solution was exchanged 5 times in a 30-min timespan to assure the complete washout of any agents before measuring a new concentration–response curve.

Multiple measurements were performed on any given specimen, however, one specimen contributed only one data set to any given concentration–response curve. To mitigate the effects of degradation of the tissue specimen over time, the order of concentration–response curve experiments was permuted between specimens. A specimen was discarded if a stable baseline tension was no longer attainable. Each concentration–response relationship shown in the figures is based on concentration–response curves from 5 specimens.

The effect of NMBAs was quantified as the depression of the force amplitude of single twitches (ST) in response to electrical stimulation of the phrenic nerve (from here on referred to as ST force amplitude). NMBA doses were given in 15-min intervals. The ST force amplitude at a given drug concentration consisted of the mean value of five consecutive contractions corrected with the baseline tension measured between contractions, measured once the contraction amplitude had stabilized and did not visibly change over time. The ST force amplitude was normalized to the maximum contraction amplitude of the untreated sample to construct cumulative concentration–response curves. Each specimen contributed 5–8 measurement points to the curve.

To determine the effect of SRBAs, a single NMBA dose was given to achieve a 90–95% depression of ST force amplitude. Then SRBA doses were given in 15-min intervals until additional doses of an SRBA were not accompanied by a further increase in ST force amplitude. A successful reversal (antagonism) was verified by measuring a TOF ratio [defined as the ratio between the fourth twitch response, T4, and the first twitch response, T1 (T4/T1) of the four stimuli] > 90%. For CMGCD curves, a reversal dose of sugammadex (0.5 mg) was given as the final dose to guarantee the full antagonism of the NMBA effect. The ST force amplitude was corrected to the ST force amplitude of the sample before administration of an SRBA and normalized to the maximum contraction amplitude after full reversal to construct cumulative concentration–response curves.

### Statistics

GraphPad Prism 6 for Windows (GraphPad Software, Inc., La Jolla, CA, USA) was used for fitting of concentration–response curves. Curve fitting was done by nonlinear regression with either the “log(agonist) vs. normalized response – variable slope,” the “log(inhibitor) vs. normalized response – variable slope,” or the “log(agonist) vs. response – Find EC anything” function. The fitting equation was: y = 100/(1 + 10^((logEC50-X)*HillSlope)), where X is the log_10_ value of concentration, and y is the normalized and baseline corrected contraction amplitude. EC_90_ and EC_99_ values were determined with the equation logEC50 = logECF—(1/HillSlope)*log(F/(100-F)), where F is 90 or 99.

For sample size determination, we performed a pilot study (n = 3) to determine the concentration–response curve of pipecuronium. We arrived at values of logEC50 = 0.14 and SD = 0.007. Assuming a 10% change in EC_50_ as clinically relevant, the group sample size at α = 0.05 and power of 80% was 5 for a two-sided test. Statistical comparison of concentration–response curves was done with GraphPad Prism 6 for Windows with the extra sum-of-squares F-test. LogEC50, LogEC90, or LogEC99 was the model component that was used to account for the extra sum-of-squares. Results are presented as mean and 95% confidence interval (CI) unless otherwise specified.

The comparison of NMBA doses used to achieve 90–95% ST depression before reversal with an SRBA and the comparison of EC_50_, EC_90,_ and EC_99_ to NMBA concentration ratios for different NMBAs was performed with Student T-test in Microsoft Excel (Microsoft Corporation, Redmond, WA, USA).

## Results

### Concentration–response relationships for different NMBAs

**Fig. 3:**
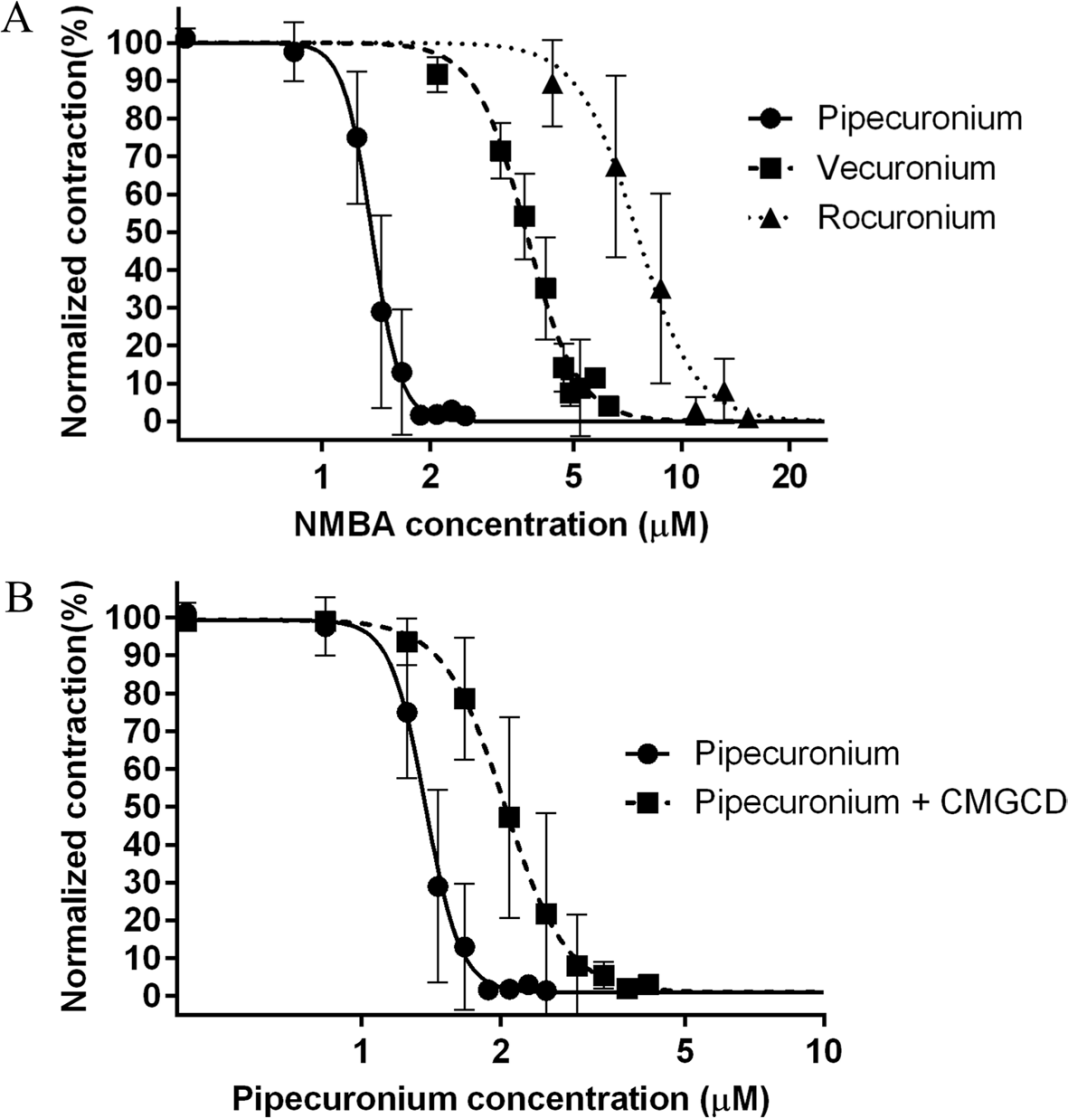
Concentration–response curves for different NMBAs*.***A** Concentration–response curves for pipecuronium, vecuronium, and rocuronium. Normalized contraction force amplitude as a function of NMBA concentration on a logarithmic scale (logarithm to base 10/ log_10_). The curve is the best fit curve calculated from measurements on n = 5 different specimens by non-linear regression. Measurement points represent mean values of normalized contraction force amplitude at a given concentration. Error bars show the standard deviation of measurement points. **B** Shift in the concentration–response curve of pipecuronium after a priming dose of 13.3 μM CMGCD. CMGCD: carboxymethyl-γ-cyclodextrin; NMBA: neuromuscular blocking agent

As a first step, we determined concentration–response curves for all investigated NMBAs (Fig. 3a). The effective concentrations for 50% (EC_50_) and 90% effect (EC_90_) are shown in Table 1. Pipecuronium displayed the greatest potency, while vecuronium had an intermediate potency. Rocuronium required the highest concentration for effective suppression of single twitch (ST) force amplitude in our ex vivo system.

**Table 1:** Comparison of EC_50_ and EC_90_ values of pipecuronium, vecuronium, and rocuronium

NMBA	EC_50_ (μM)	EC_90_ (μM)
Pipecuronium	**1.38** (1.33–1.42)	**1.68** (1.58–1.79)
Pipecuronium + CMGCD	**2.04** (1.94–2.16)	**2.90** (2.58–3.25)
Vecuronium	**3.69** (3.59–3.80)	**5.29** (4.97–5.63)
Rocuronium	**7.50** (6.93–8.12)	**11.36** (9.64–13.39)

Values are given as mean and 95% confidence interval. EC_50_, effective concentration for 50% effect; EC_90_, effective concentration for 90% effect; Pipecuronium + CMGCD, pipecuronium effective concentrations after a priming dose of 13.3 μM carboxymethyl-γ-cyclodextrin. *CMGCD* Carboxymethyl-γ-cyclodextrin

### Mechanism of NMBA block reversal with CMGCD

To evaluate the nature of the interaction between CMGCD and NMBAs, CMGCD was administered in a concentration of 13.3 μM to the buffer solution before incremental dosing of pipecuronium. Pre-treatment with CMGCD resulted in a significant right-shift of the dose–response curve of pipecuronium (Fig. 3b). However, maximum ST depression could still be achieved in the presence of CMGCD, although at a higher pipecuronium concentration (compared to when CMGCD was not present). This indicates the capture of pipecuronium by CMGCD until its binding capacity was saturated, after which the unbound NMBA molecules induced ST depression.

### The potency of CMGCDs for reversal of neuromuscular block compared to sugammadex

**Fig. 4:**
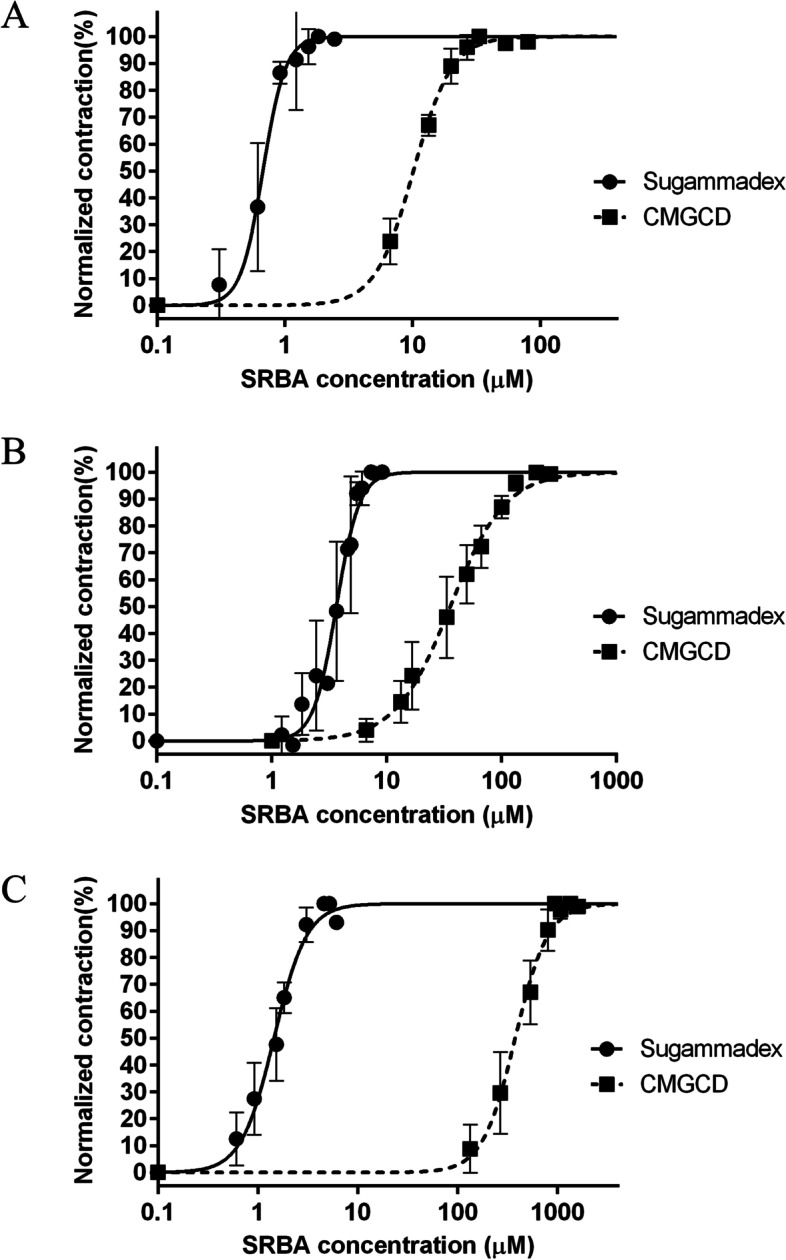
Concentration–response curves for SRBAs. Concentration–response curves for CMGCD and sugammadex for reversing (**A**) pipecuronium-induced, (**B**) rocuronium-induced, (**C**) and vecuronium-induced neuromuscular block. Normalized contraction force amplitude as a function of SRBA concentration on a logarithmic scale (logarithm to base 10/ log_10_). The curve is the best fit curve calculated from measurements on n = 5 different specimens by non-linear regression. Measurement points represent mean values of normalized contraction force amplitude at a given concentration. Error bars show the standard deviation of measurement points. CMGCD: carboxymethyl-γ-cyclodextrin; SRBA: selective relaxant binding agent

The concentration–response curves of CMGCD and sugammadex were determined for each of the three NMBAs (Fig. 4). The EC_50_ and EC_90_ values of sugammadex and CMGCD were the lowest with pipecuronium (Table 2). Whereas potency of sugammadex to reverse vecuronium-induced block was greater than for rocuronium-induced block, CMGCD displayed greater potency for rocuronium and was least effective against vecuronium. With any given NMBA, sugammadex had significantly lower EC_50_ and EC_90_ values than CMGCD (*p* < 0.0001). Whereas CMGCD achieved full reversal of pipecuronium-induced neuromuscular block, 94.6 ± 1.5% and 86.3 ± 5% of maximum ST force amplitude were achieved in the case of rocuronium and vecuronium, respectively. Interestingly, the return of ST force amplitude from rocuronium-induced block was greater than for the TOF ratio (94.6% vs 90.6%, respectively), while for vecuronium the recovery of ST force amplitude was smaller than the recovery of the TOF ratio (86.3% vs 87.6%, respectively). However, the difference between ST return and TOF ratio was statistically non-significant for both NMBAs (*p* = 0.249 and *p* = 0.637 for rocuronium and vecuronium, respectively).

**Table 2:** Comparison of EC_50_ and EC_90_ values of CMGCD and sugammadex

NMBA	SRBA	EC_50_ (μM)	EC_90_ (μM)
**Rocuronium**	**CMGCD**	**35.89** (32.67–39.41)	**119.9** (101.5–141.9)
	**Sugammadex**	**3.67** (3.43–3.92)	**6.08** (5.34–6.92)
**Pipecuronium**	**CMGCD**	**10.14** (9.61–10.70)	**21.60** (19.63–23.76)
	**Sugammadex**	**0.67** (0.62–0.74)	**1.05** (0.86–1.29)
**Vecuronium**	**CMGCD**	**376.1** (341.9–413.8)	**863.1** (727.8–1023)
	**Sugammadex**	**1.45** (1.35–1.56)	**3.33** (2.82–3.92)

Values are given as mean and 95% confidence interval. EC_50_, effective concentration for 50% effect; EC_90_, effective concentration for 90% effect. *CMGCD* Carboxymethyl-γ-cyclodextrin

### The affinity of SRBAs for NMBAs

Since NMBAs were administered to achieve at least 90% twitch amplitude depression, the differences in NMBA potency resulted in significantly different NMBA doses (*p* < 0.0001 for all NMBA pairs). To better visualize the actual SRBA affinity for a given NMBA, for each SRBA we calculated the ratio of the EC_50_ and EC_90_ values to the dose of the NMBA applied, yielding corrected effective concentration values (EC_50,dc_ and EC_90,dc_; Table 3). For sugammadex, there was no statistical difference between these corrected effective doses of pipecuronium, rocuronium, or vecuronium. The dose–response curves for sugammadex were very steep, therefore we also calculated the EC_99_ to NMBA dose ratio. The EC_99,dc_ value for vecuronium was significantly higher than for pipecuronium (*p* = 0.024) or rocuronium (*p* = 0.013). In the case of CMGCD, EC_50,dc_ and EC_90,dc_ values were significantly higher for vecuronium than for the other two NMBAs. The EC_50,dc_ and EC_90,dc_ of rocuronium were non-significantly lower than those of pipecuronium.

**Table 3:** Comparison of EC_50,dc_, EC_90,dc_ and EC_99,dc_ values of CMGCD and sugammadex

NMBA	SRBA	EC_50,dc_ (μM)	EC_90,dc_ (μM)	EC_99,dc_ (μM)
**Rocuronium**	**CMGCD**	**3.81** (2.44–5.19)	**10.60** (9.63–11.57)	**-**
	**Sugammadex**	**0.34** (0.24–0.44)	**0.55** (0.48–0.62)	**0.95** (0.82–1.08)
**Pipecuronium**	**CMGCD**	**5.15** (4.21–6.08)	**11.02** (8.95–13.08)	**-**
	**Sugammadex**	**0.38** (0.23–0.54)	**0.58** (0.43–0.74)	**0.95** (0.69–1.20)
**Vecuronium**	**CMGCD**	**101.2** (73.9–128.4)	**306.0** (172.7–439.2)	**-**
	**Sugammadex**	**0.32** (0.23–0.41)	**0.68** (0.54–0.83)	**1.67** (1.08–2.26)

Values are given as mean and 95% confidence interval. EC_50,dc_, EC_90,dc_ and EC_99,dc_, effective concentrations for 50, 90, and 99% effect, respectively, of relaxant binding agents normalized to the neuromuscular blocking agent concentration. *CMGCD* Carboxymethyl-γ-cyclodextrin

## Discussion

In this ex vivo study, we investigated the reversal of rocuronium-, pipecuronium-, and vecuronium-induced neuromuscular block with a novel SRBA, carboxymethyl-γ-cyclodextrin. The potency of CMGCD to reverse neuromuscular block was greatest for pipecuronium, followed by rocuronium, and lastly vecuronium. The potency of CMGCD was lower than that of sugammadex. CMGCD, just as sugammadex, displayed a similar affinity for pipecuronium and rocuronium, but bound vecuronium less efficiently.

A fundamental part of anaesthesia care, muscle relaxation also has the potential to cause significant harm if still present after the extubation of the trachea [19, 20]. Concerns over the postoperative residual neuromuscular block (PORNB) and the side-effects of acetylcholinesterase inhibitors led to a shift in clinical practice from long-acting to intermediate-acting NMBAs. However, PORNB is not reliably eliminated by using intermediate-duration NMBAs [21], therefore routine monitoring of neuromuscular block and adequate reversal, if needed, are a cornerstone of ensuring patient safety. Selective relaxant binding agents, and more specifically sugammadex, allow for the fast and reliable reversal of neuromuscular block with minimal side effects [8].

Although rare, allergic reactions and anaphylaxis in both adult [22] and paediatric [23] patients continue to be reported. Other very rare, but potentially significant, side effects associated with the use of sugammadex include atropine-resistant bradycardia and asystole [24, 25]. Additionally, sugammadex is ineffective against benzylisoquinoline NMBAs such as atracurium or cisatracurium [26]. It is therefore not surprising that there is a constant search for alternative NMBA antagonists that may improve on the reversal spectrum and reduce the potential for clinically relevant side effects.

To test the efficacy of SRBAs to reverse neuromuscular block, we used an isolated tissue preparation consisting of rat hemidiaphragm and associated phrenic nerve. Originally described by Bülbring in the 1930s [18], the hemidiaphragm-phrenic nerve specimen was used in several classical experiments that helped elucidate the pharmacology of the neuromuscular junction [27, 28]. Since the normal function of the neuromuscular junction and electro-mechanical coupling following nerve stimulation is maintained in the hemidiaphragm specimen, this ex vivo system is a valuable screening tool that allows the collection of clinically relevant data without having to perform actual live animal experiments.

We constructed concentration–response curves and determined the EC_50_ values for three aminosteroidal NMBAs in clinical use: rocuronium, pipecuronium, and vecuronium. These EC_50_ values were in good agreement with previously published values measured in a similar system [29], supporting the validity of our experimental setup. Pipecuronium showed the greatest potency, followed by vecuronium and rocuronium.

In the present study, both CMGCD and sugammadex exhibited the ability to antagonize the neuromuscular block induced by rocuronium, pipecuronium, and vecuronium. The dose-corrected EC_50_ and EC_90_ values were not statistically different between rocuronium and pipecuronium. In the case of vecuronium, CMGCD potency for reversal was low, with comparably higher EC_50_ and EC_90_ values, even after dose correction. The efficacy of CMGCD for reversal varied according to the NMBA used, as evidenced by the increasing contraction force amplitude that resulted from additional sugammadex administration in the case of rocuronium and vecuronium.

Originally marketed for the reversal of rocuronium-induced neuromuscular block, sugammadex has since been shown to be clinically effective against pipecuronium- and vecuronium-induced block as well [12, 30]. Our results are in line with these clinical findings, as sugammadex showed a similar affinity for pipecuronium, rocuronium, and vecuronium when considering corrected EC_50_ and EC_90_ values and a decreased affinity for vecuronium with corrected EC_99_ values. The ratio of EC_99_ to NMBA dose was approximately 1 for pipecuronium and rocuronium, meaning that an equimolar dose of sugammadex is sufficient for a full recovery of the twitch height after the reversal of the neuromuscular block induced with these NMBAs. For vecuronium, this ratio was closer to 1.7, implying a reduced binding efficacy, in which nearly twice as many sugammadex molecules are needed to bind all NMBAs.

The limits of our study arise from its ex vivo nature. Pharmacokinetics are eliminated in our model system, therefore actual block dynamics can be different in vivo. We used ST stimulation to monitor neuromuscular function, which is less sensitive than the TOF ratio used in the clinical setting. Additionally, the relationship between the fade of the TOF ratio and ST-depression can be agent-specific [31, 32]. Lastly, the in vitro concentration–response data are not necessarily indicative of the different types of responses (time to recovery, reparalysis events, etc.) measured in clinical studies.

## Conclusion

We conclude, that carboxymethyl-γ-cyclodextrin (CMGCD) is an effective agent to reverse rocuronium- and pipecuronium-induced neuromuscular block ex vivo. Although CMGCD shows a 15–20-fold decrease in potency in comparison with sugammadex, this can be compensated by the administration of higher doses. Further research is warranted for optimizations in the substitution profile of CMGCD to help increase affinity and reduce dose requirements.

## Data Availability

The datasets used and/or analysed during the current study are available from the corresponding author on reasonable request.

## Abbreviations

NMBA: Neuromuscular blocking agent
TOF: Train-of-four
SRBA: Selective relaxant binding agent
CMGCD: Carboxymethyl-γ-cyclodextrin
ST: Single twitch
